# An Approach for Autonomous Feeding Robot Path Planning in Poultry Smart Farm

**DOI:** 10.3390/ani12223089

**Published:** 2022-11-09

**Authors:** Yanjun Zhang, Weiming Sun, Jian Yang, Weiwei Wu, Hong Miao, Shanwen Zhang

**Affiliations:** College of Mechanical Engineering, Yangzhou University, Yangzhou 225127, China

**Keywords:** smart farms, precision agriculture, animal welfare, feeding robot, path planning, branch and bound, genetic algorithm

## Abstract

**Simple Summary:**

In the poultry feeding process, a feeding robot instead of manual feed delivery can solve the problems of high labor demand and untimely feeding. However, if the feeding robot is not guaranteed to travel in an optimal path, it will cause large amounts of unnecessary energy consumption. In order to obtain the minimum energy consumption travel path of the feeding robot, the energy consumption of the feeding robot was taken as the optimization objective of the path planning in this study, and the minimum energy consumption travel path planning algorithm was designed. The experiment results show that the minimum energy consumption travel path could be obtained by the algorithms proposed in this study at the specified time, and that they have more computing power. The methods proposed in this study can reduce the production cost of the poultry smart farm to a certain extent and promote the development of poultry smart farms.

**Abstract:**

In order to solve the problems of poor feeding environment, untimely feeding and high labor demand in poultry smart farms, the development of feeding robots is imminent, while the research on path planning algorithms is an important part of developing feeding robots. The energy consumption of the feeding robot is one of the important elements of concern in the process of path planning. In this study, the shortest path does not mean that the feeding robot consumes the least energy, because the total mass of the feeding robot keeps changing during the feeding process. It is necessary to find the most suitable path so that the feeding robot consumes the lowest amount of energy during the feeding process. A branch and bound algorithm to calculate the minimum energy consumption travel path for small-scale buckets lacking feed is proposed. The lower bound of the branch and bound on the energy consumption is obtained by the approach of preferred selection of the set of shortest edges combined with the sequence inequality, and the upper bound could be obtained based on Christofides’s Heuristic algorithm. A double-crossover operator genetic algorithm based on an upper bound on energy consumption for large-scale buckets lacking feed is proposed, and different crossover operations are performed according to the relationship between the fitness value and the upper bound of energy consumption in order to find a better path. The experiment results show that the approach proposed in this study is efficient; for small-scale buckets lacking feed, a branch and bound algorithm could calculate the minimum energy consumption path of 17 points in 300 s, and for large-scale buckets lacking feed, a double-crossover operator genetic algorithm based on an upper bound on energy consumption could calculate the minimum energy consumption travel path within 30 points in 60 s. The result is more accurate compared to the genetic algorithm with a single crossover operator.

## 1. Introduction

Animal welfare farming is gradually becoming the main method for smart farms. The purpose of welfare farming is to create additional value for animals and consumers by helping farms resolve the conflict between animal welfare and efficient farming, as well as by supporting reduced production intensity and intensive farming [1]. Intensive farming, as a common global agricultural production approach, has met the increasing human demand for meat-protein dairy products, but the survival and health of farm animals in this mode of farming are seriously neglected [2], and their meat quality is lower compared to semi-intensive farming [3]. Semi-intensive farming is usually conducted outdoors under non-structural conditions, with the aim of providing animals with a free and comfortable growing environment. However, large-scale farming under non-structural conditions has a high demand for labor: the data show that the percentage of agricultural labor participation in 2017 was 1.66%, 1.28%, 3.49% and 6.70% in the United States, Japan, Germany and Russia [4]. This is still a decreasing trend year by year, resulting in a lower and lower agricultural labor force in the future, and the demand for “machine for human” is increasing. In recent years, with the rapid development of new generation information technology such as Internet of Things [5,6], big data [7], artificial intelligence [8,9] and intelligent equipment manufacturing [10,11], these technologies will gradually be widely applied to all aspects of agricultural production. The smart farm could realize information perception, quantitative decision-making, intelligent control, precise input and personalized service in the entire process of agricultural production and management [12].

Research on and development of feeding robots could fundamentally solve issues such as the feeding labor shortage, high labor intensity and untimely feeding. Currently, there is more research on feeding robots for structured poultry and livestock enclosures (e.g., cattle barns, sheep barns, etc.). The sheep feeder SF60, a stationary automatic feeding robot designed by BioControl AS in the Oslo, Norway, consists mainly of front and rear doors, sheep feeding troughs, feed bins and controllers. The sheep feeder SF60 is capable of providing concentrated feed to sheep according to the settings made by the user and by reading the RFID ear tag information [13]. The Triloliet HP 2300 automatic feeding device designed by Trioliet B.V. in Rotterdam, Netherlands, is a suspended, self-contained feeding robot consisting of a 3 m^3^ hopper, two vertical churns, discharge belts on both sides, am infinitely adjustable speed control belt and a mixing enhancer, which acts as a fully automatic feeding system in conjunction with a stationary feed mixer [14]. The aerial conveyor feeding system, produced by PELLON in Tampere, Finland, consists of filler unit and concentrate tower to put the feed group into the fixed feed mixing device. The mixed feed is sent to the feed conveyor by the lifting conveyor, and under the thrust of the sliding plow device on the conveyor, it is evenly sprinkled on the feeding surface to complete the feeding operation [15].

The aforementioned research on feeding robots is mainly for intensive farms. Semi-intensive farms are generally flatland free-range farms that cover a large area. A large number of feed buckets are placed in the site, and a large amount of manual transportation and feeding is required. This kind of feeding mode is relatively crude; it is difficult to be quantitative and accurate, and often there is not enough feed in the buckets, which leads to some livestock and poultry having no feed to eat or overfeeding. At the same time, the process of transporting and feeding is labor-intensive and the operating environment is poor.

According to the above review of the development of the feeding robots, current typical automatic feeding devices use magnetic guidance or laser-guided techniques as their strategies for travel in structured farming environment. These control strategies cannot be used in the unstructured farming environment in this paper. This is because the feed buckets are scattered throughout the farm in semi-intensive farms; the activity areas of livestock and poultry are uncertain; the number of feeding buckets lacking feed is different during each feeding; and some feeding buckets need feeding, while others do not, which leads the feeding robot to work in different paths each time. Thus, the above robot control strategies are not applicable to this study. The key to path planning is the traversal order of the feeding robot, which is equivalent to a multi-objective path planning problem. Research on path planning problems with multiple objective points has focused on the study of algorithms; accurate algorithms are generally used to find the exact path of small-scale target points. Wang et al. [16] proposed a tailored branch and bound algorithm to obtain near-optimal solutions in small-scale instances in automated container terminals. Meneguzzi et al. [17] used the branch and bound algorithm to solve the path planning model in order to obtain the forest inventory vehicles path. Thakar et al. [18] presented a branch and bound-based algorithm to determine spray paths on a point cloud of the surface being disinfected. From the above review, it is clear that branch and bound has been applied to solve many problems in engineering, but the above applications for branch and bound algorithms are dominated by the path length and do not involve energy consumption. The exact algorithm (e.g., branch and bound) is computationally inefficient and cannot calculate the path of large-scale target points, so the optimal path can only be calculated by heuristic algorithm. Xie et al. [19] proposed an improved ant colony optimization algorithm to solve the multi-objective detection path planning for radioactive environments, which not only combines the ant colony algorithm and chaos optimization algorithm, but also introduces the pheromone difference update strategy and local search optimization strategy to obtain a more reasonable detection path. Multi-objective path planning for agricultural robots under virtual greenhouse conditions by Mahmud et al. [20], a reference point-based non-dominated sorting genetic algorithm, was used to determine the best path (NSGA-III), which was then compared with the non-dominated sorting genetic algorithm (NSGA-II), and the quality of the results was verified. Zacharia et al. [21] proposed a minimum time path planning strategy for the multi-point manufacturing problem in drilling/spot welding tasks, transforming the problem into a travel salesman problem and applying a genetic algorithm in order to obtain the final path. Although the heuristic algorithm can solve the problem at a faster rate, its solution result is an approximation of the best result, and the above studies are length-driven for solving, unrelated to energy consumption.

For the solution of multi-objective point path planning-related problems, the path length is generally taken as the dominant solution [22]. For smart farms, the energy consumption of feeding robots is very important, and reducing the energy consumption of feeding robots will reduce the operation cost of unmanned farms to a certain extent. Therefore, this study will take energy consumption as the optimization objective to solve the multi-objective path planning problem with a variable number of target points.

In this paper, we first describe and analyze the problem under study, determine the conditions for triggering robot path planning and divide the number of feed buckets in the problem into different scales in order to find the minimum energy consumption path separately. Then, the methods for solving problems in this study are proposed. For the small-scale feed buckets lacking feed, the travel path with minimum energy consumption is obtained by branch and bound algorithm. For the large-scale feed buckets lacking feed, the travel path with minimum energy consumption approximation is obtained by double-crossing operator genetic algorithm based on the upper bound of energy consumption. Finally, the effectiveness of the algorithms proposed in this study is verified through comparative experiments.

## 2. Materials and Methods

### 2.1. Problem Description and Analysis

**Figure 1:**
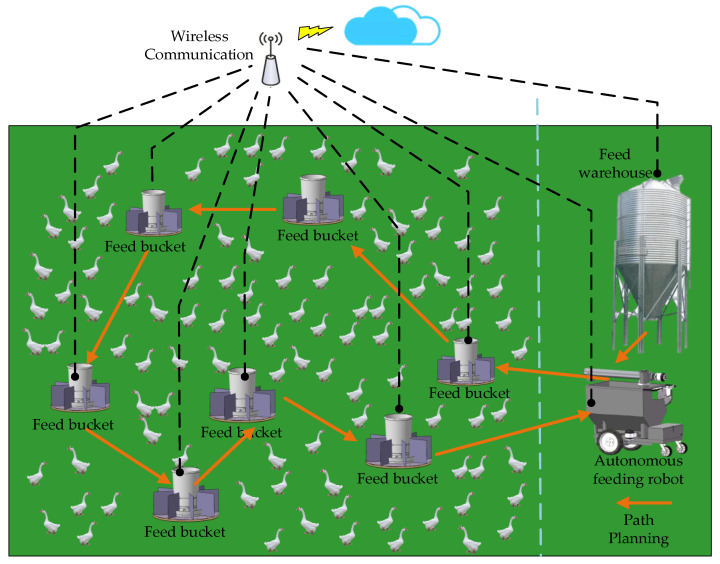
An example of a poultry (goose) smart farm.

We can see from the simulated smart farm in Figure 1 that there are *n* feed buckets distributed in the smart farm, and the feed buckets are equipped with weighing sensors and wireless communication modules. The feeding robot can receive signals of the remaining amount of feed in each bucket in real time. When the weight of feed in each bucket meets the conditions set, the robot starts to plan the path to complete the feeding process independently.

In this study, the shortest path does not mean that the feeding robot consumes the least energy, because the total mass of the feeding robot changes during the feeding process. In addition, the number of buckets to be fed at a time is uncertain. When the weight of feed in the bucket is below the threshold set, it will be brought into the target set for the feeding robot’s path planning. According to the number of buckets short of feed and the shortage of feed for each bucket, the conditions for triggering robot path planning are divided into two cases. The first one is urgent feeding depending on the situation, and the second one is feeding at the regular time.

**Figure 2:**
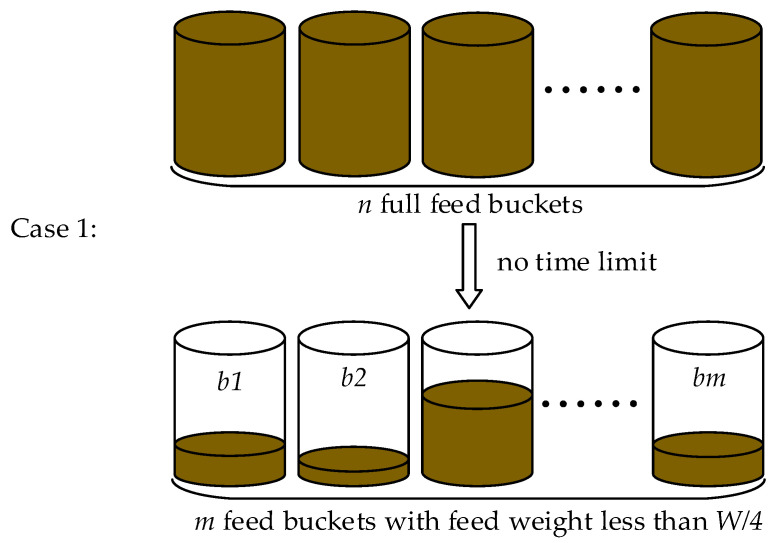
Case 1 for the robot to start feeding.

Case 1: The urgent feeding case is shown in Figure 2, depending on the situation, is that the feeding robot starts path planning and feeding when there are *m* buckets in the farm with less than *W*/4 of feed, where *W* is the feed weight when the buckets are full and the relationship between *n* and *m* is Equation (1).

(1) $$m=\frac{2}{3}n$$

**Figure 3:**
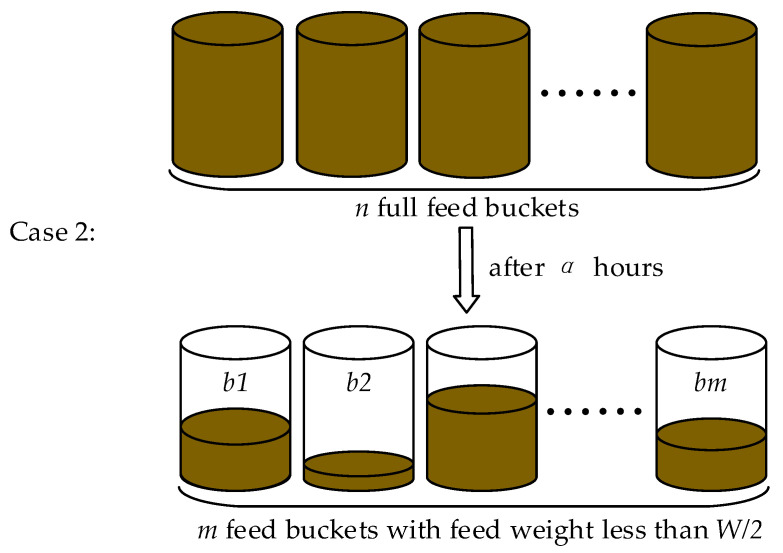
Case 2 for the robot to start feeding.

Case 2: The case of feeding at the regular time is shown in Figure 3 is that the feeding robot starts path planning and feeding when there are m feed buckets more than z feed buckets with feed weight less than *W*/2, and after α hours since the last feeding time (z is the threshold).

**Figure 4:**
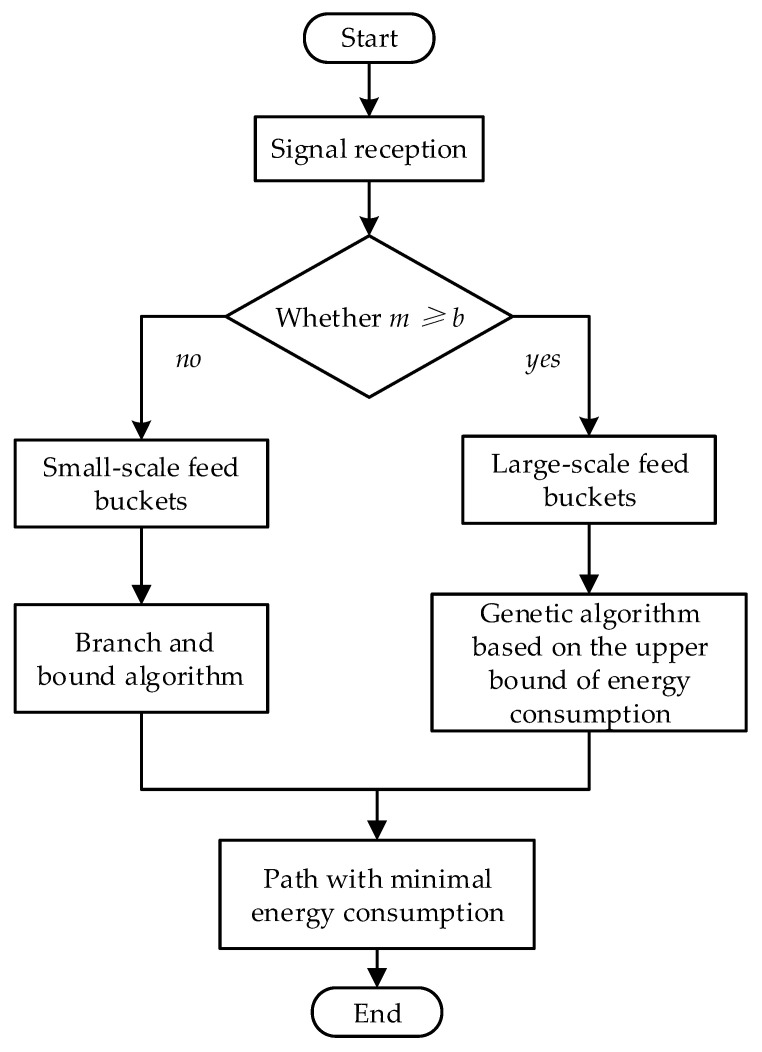
The main flow of the approach in this study.

The main process of the approach is shown in Figure 4, and each feed bucket is regarded as a point in the path planning algorithm for the feeding robot. b is the boundary value of the small scale and the large scale of feed points. The energy consumption used by the feeding robot to complete the feeding task is the optimization objective. For a small scale of feed points, the branch and bound algorithm is proposed for path planning. The genetic algorithm based on the upper bound of energy consumption is proposed for a large scale of feed points.

### 2.2. Path Planning for Small-Scale Feeding Point

#### 2.2.1. Theory of the Branch and Bound Algorithm

For the path planning of the small-scale feeding points, the exact algorithm or the heuristic algorithm could be used. Generally, the exact algorithm could obtain an optimal solution but takes more time, while the heuristic algorithm could obtain an approximation of the optimal solution in less time [23]. The minimum energy consumption path of the feeding robot could be obtained by the exact algorithm within an acceptable calculation time. Therefore, the branch and bound algorithm, a kind of exact algorithm, is used to calculate the minimum energy consumption for small-scale feeding point path planning.

The start point of the feeding robot is regarded as the root point of the search tree of the branch and bound algorithm. The process of branching is to add child points to the tree, and the bounding is to check the values of the upper and lower bounds of energy consumption with the added points until the optimal solution is obtained.

#### 2.2.2. Boundary Constraints of Branch and Bound Algorithms

The time of the branch and bound algorithm taken is one of the important indexes to evaluate performance of the algorithm, and it is dependent on the method to determine the upper and lower bounds [24]. For the path planning of small-scale feeding points, the approach of preferred selection of the set of shortest edges combined with the sequence inequality is proposed to calculate the lower bound, and the upper bound could be obtained based on Christofides’s heuristic algorithm.

#### 2.2.3. Solution of the Global Lower Bound

In general, the lower bound (*LB*) is used to find the optimal solution in the search tree, and the point with the minimum value of *LB* in the search tree would be expanded each time. A more accurate *LB* is conducive to finding the optimal solution. In order to improve the efficiency of the algorithm, an approach to computing the *LB* is proposed, which is called preferred selection of the set of shortest edges combined with the sequence inequality.

A complete graph *G = (V*, *E)* is defined, where $V=\left\{v_{0},v_{1},v_{2},\cdots ,v_{n}\right\}$, *v*_0_ is the starting and the final return point of the feeding robot, *v*_1_, *v*_2_,…, *v_n_* represents the set of feed buckets in the farm and *E* is the set of edges of the complete graph *G*. Any two points are connected by an edge. For any edge in *G*, the path from *v_i_* to *v_j_* is *e_ij_*. The path of the feeding robot satisfies the symmetric property that the distance between any two feeding points satisfies *e_ij_ = e_ji_* (*i*, *j* ∈ *V*). Thus, the distance between any two points can be expressed as the following symmetric matrix.

$E=\left|\begin{array}{cccccc} - & e_{01} & \cdots & e_{0i} & \cdots & e_{0n}\\ e_{10} & - & \cdots & e_{1i} & \cdots & \vdots \\ \vdots & \vdots & - & \vdots & \cdots & \vdots \\ e_{i0} & e_{i1} & \cdots & - & \cdots & e_{in}\\ \vdots & \vdots & \vdots & \vdots & - & \vdots \\ e_{n0} & \cdots & \cdots & e_{ni} & \cdots & - \end{array}\right|$, where $i,j\in \left\{0,1,2,\cdots ,n\right\}$.

For the bucket to require feeding, a complete graph *G_m_* = (*V*_0_, *E*_0_) could be defined: $V_{0}=\left\{v_{0},v_{1},v_{2},\cdots ,v_{m}\right\}$, *v*_0_ is the starting and the final return point of the feeding robot, $V_{1}=\left\{v_{1},v_{2},\cdots ,v_{m}\right\}$ represents the set of feed buckets in the farm and the weight of feed required for each bucket is $F=\left\{q_{1},q_{2},\cdots ,q_{m}\right\}$. *E*_0_ is the set of edges of the complete graph *G_m_*. The robot’s path is represented by the following: $B=\left\{e_{01},e_{12},\cdots ,e_{ij},\cdots ,e_{(m-1)\begin{array}{c} m \end{array}},e_{m0}\right\}$, where *e_ij_* is the edge from *v_i_* to *v_j_* and the length of *e_ij_* is *d_i_*. The energy consumed by the feeding robot for the path *B* could be expressed as Equation (2):

(2) $$C\left(B\right)=d_{0}(w+Q)+d_{1}\left(w+Q-q_{1}\right)+\cdots +d_{{}_{m-1}}\left(w+Q-\sum_{i=1}^{m-1}q_{i}\right)+d_{m}w$$

where *w* is the net weight of the feeding robot and *Q* is the total weight of the required feed. For the complete graph *G_m_*, there are *m* edges connected to *v_i_*. From the path *B*, it is known that the feeding robot needs to pass through *m* + 1 points and *m* + 1 edges to finish the feeding task. In order to find the lower bound of the energy consumption of the feeding robot, it is necessary to find the lower bound by choosing *m* + 1 suitable and relatively short edges. The approach of preferred selection of the set of shortest edges is as follows:

Firstly, create the set *H* of shortest edges, which could contain only *m +* 1 elements. Then, choose the shortest edge *h_i_* from the *m* edges corresponding to each point as an edge in set *H*. The shortest edges could be chosen from the distance matrix *E*, the minimum value of each row is the shortest edge corresponding to the point and the edges chosen are then added to the set *H*. To further select the most suitable shortest edge, duplicate selection of edges is prohibited. Since *e_ij_* and *e_ji_* are the same edge, when the shortest edge *e_ij_* of an edge point *v_i_* ∈ *V*_1_ is selected, *e_ji_* cannot be selected as the shortest edge again. Therefore, if the shortest edge of point *v_j_* is *e_ij_* and *e_ji_* has been selected, the secondary short edge in the two set of edges corresponding to *v_i_* and *v_j_* is added to set *H*. Finally, $H=\left\{h_{0},h_{1},\cdots ,h_{m}\right\}$ is obtained, and it could be expressed as an increasing sequence matrix $H^{*}=\left[h_{0}^{*},h_{1}^{*},\cdots ,h_{m}^{*}\right]$.

In order to facilitate the description of the function in Equation (2), *C*(*B*) could be expressed as:

(3) $$C\left(B\right)=A_{}^{T}\cdot M$$

In Equation (3), the sequence matrix, composed of the lengths of edges the feeding robot passed through, is $A={\left[d_{0},d_{1},\cdots ,d_{m}\right]}^{T}$. The sequence matrix composed of the total weight of the feeding robot, corresponding to edges the feeding robot passed through, is $M={\left[w+Q,w+Q-q_{1},\cdots ,w\right]}^{T}$. In order to minimize *C*(*B*) to find *LB*, the two sequences should be arranged in opposite ways based on the sequence inequality theory [25]. The sequence in *M* is decreasingly aligned, and each element in the sequence has three constants, *w*, *Q* and *q*. For the *q* in the sequence elements, we could decrease rank sequence *F* in order to construct a completely decreasing sequence matrix:

(4) $$M^{*}=\left[w+Q,w+Q-q_{1}^{*},\cdots ,w+Q-\sum_{i=1}^{m-1}q_{i}^{*},w\right]$$

The decreasing sequence F* is $\left\{q_{0}^{*},q_{1}^{*},\cdots ,q_{m}^{*}\right\}$, $\left(q_{0}^{*}=0\right)$. Thus, for any path *B*, we have the following relation:

(5) $$C\left(B\right)=A_{}^{T}\cdot M\geq A_{}^{T}\cdot M^{*}$$

The edge length increasing sequence matrix of *A* is $A^{*}=\left[d_{0}^{*},d_{1}^{*},\cdots ,d_{m}^{*}\right]$. Based on the sequence inequality theory, for any path *B*, we have the following relation:

(6) $$C\left(B\right)=A_{}^{T}\cdot M\geq {\left(A^{*}\right)}_{}^{T}\cdot M^{*}$$

Then, (A^*^)^T^·M^*^ is the lower bound of the current path, not the lower bound of all paths. For a complete work process of the feeding robot, regardless of how the feeding robot plans the path, the weight of the feed bucket shortage is fixed, so M^*^ in Equation (4) is determined. To find the lower bound of energy consumption of all paths, we need to find the relatively short edge sequence; for each item within A^*^ and each item in H^*^, there is such a relationship:

(7) $$d_{i}^{*}\geq h_{i}^{*},d_{i}^{*}\in A^{*}\begin{array}{c} and \end{array}\begin{array}{c} h_{i}^{*}\in H^{*} \end{array}$$

The reason is that when the edge *h_i_* in the set *H* is shortest corresponding to *v_i_* (i.e., *e_ij_* is the shortest edge in the set corresponding to *v_i_*, and *e_ij_* is not shortest corresponding to *v_j_*), and *d_i_* is one of the edges in the set *A* corresponding to *v_i_*, so for each element of the same index number in both sequences, there is *d_i_* ≥ *h_i_*. When there exist edges in the set *A* that are the shortest edges of their corresponding points, and the edges corresponding to these points in the set *H* are also the shortest edges, it is obvious that *d_i_* ≥ *h_i_*. Sometimes, there exist edges in set *A* that are the shortest edges of their corresponding points, and these edges in set *H* are not the shortest edges of their corresponding points, i.e., *e_ij_* in set *A* is the shortest edge (*d_i_ = e_ij_*) corresponding to point *v_i_*, but in set *H*, *e_ij_* is not the shortest edge corresponding to point *v_i_*, and *e_ji_* must be the shortest edge corresponding to *v_j_*, i.e., *d_i_ = e_ji_*. Therefore, although *d_i_* < *h_i_*, *d_j_* ≥ *h_i_* and *d_i_* = *h_j_* must also be true.

From Equation (7), it is known that for any path *B* there is the following relation:

(8) $$C\left(B\right)={\left(A\right)}^{T}\cdot M\geq {\left(A^{*}\right)}^{T}\cdot M^{*}\geq {\left(H^{*}\right)}^{T}\cdot M^{*}$$

Therefore, the lower bound on the energy consumed by the feeding robot to complete the entire feeding task is given by Equation (9):

(9) $$LB={\left(H^{*}\right)}^{T}\cdot M^{*}$$

#### 2.2.4. Solution of the Partial Lower Bound for the Energy Consumption

In the process of branch and bound, the path points could be divided into determined path points and undetermined path points; for example, assume a path has 7 points (it include the starting point), the determined path of the feeding robot is $\left\{e_{05},e_{52},e_{23}\right\}$ and the path which will pass points *v*_1_, *v*_4_ and *v*_6_ is undetermined. If the global *LB* is still used for the process of branching and bounding, the efficiency of the branch and bound algorithm will be greatly reduced, so the partial lower bound (*PLB*) for possible paths need to be calculated.

The calculation of *PLB* is divided into two parts. The first part is the calculation of energy consumption *C*(*B_k_*) of the determined path, and the second part is the calculation of lower bound *LB_m−k_* of the energy consumption of the undetermined path, so the *PLB* could be expressed as Equation (10):

(10) $$PLB=C(B_{k})+LB_{m-k}$$

In order to facilitate the description of the calculation, assume that the set of path points of the determined path is $V_{k}=\left\{v_{0},v_{1},\cdots ,v_{k}\right\}$ ($V_{k}\in V_{m}$), and the partial determined path is $B_{k}=\left\{e_{01},e_{12},\cdots ,e_{(k-1)k}\right\}$. *B_k_* contains at least two points (i.e., *k* ≥ 1), and the sequence of the weight of feed required for each bucket is $F_{k}=\left\{q_{0},q_{1},\cdots ,q_{k}\right\}$. The *C*(*B_k_*) could be calculated by Equation (11):

(11) $$C\left(B_{k}\right)=d_{0}(w+Q)+d_{1}(w+Q-q_{1})+\cdots +d_{k-1}(w+Q-\sum_{i=1}^{k-1}q_{i})$$

The calculation of the undetermined path is same as that of *LB* for the total path. The undetermined path is $B_{m-k}=\left\{e_{k(k+1)},\cdots ,e_{(m-1)m},e_{m0}\right\}$, the decreasing sequence of the feed bucket missing weight is $F_{m-k}^{*}=\left\{q_{k}^{*},q_{k+1}^{*},\cdots ,q_{m}^{*}\right\}$ and the decreasing sequence matrix consisting of the total weight of the feeding robot on the corresponding edges is as follows in Equation (12):

(12) $$M_{m-k}^{*}={\left[w+Q-\sum_{i=1}^{k}q_{{}_{i}}^{*},w+Q-\sum_{i=1}^{k+1}q_{{}_{i}}^{*},\cdots ,w+Q-\sum_{i=1}^{m-1}q_{{}_{i}}^{*},w+Q-\sum_{i=1}^{m}q_{{}_{i}}^{*}\right]}^{T}$$

The sequence matrix of undetermined path points corresponding to the set of shortest edges is $H_{m-k}^{}={\left[h_{k}^{},h_{k+1}^{},\cdots ,h_{m-1}^{},h_{m}^{}\right]}^{T}$, and the decreasing sequence matrix of the *H_m−k_* is $H_{m-k}^{*}={\left[h_{k}^{*},h_{k+1}^{*},\cdots ,h_{m-1}^{*},h_{m}^{*}\right]}^{T}$. Therefore, the *LB_m−k_* could be calculated by Equation (13):

(13) $$LB_{m-k}={\left(H_{m-k}^{*}\right)}^{T}\cdot M_{m-k}^{*}$$

#### 2.2.5. Solution of the Upper Bound of Energy Consumption

For branch and bound algorithms, there is generally only one value of upper bound *UB* in the search process. When the value of the lower bound of any point is larger than the value of *UB*, the point cannot be extended, and when the value of energy consumption of the complete path is less than the value of *UB*, the upper bound is updated to the current value of optimal path energy consumption.

The *UB* for the energy consumption of the feeding robot will be obtained based on Christofides’s heuristic algorithm [26]. Christofides’s heuristic algorithm is an approximation algorithm for combinatorial optimization problems with an approximation ratio of 3/2, which is the best approximation ratio in the general metric space. The bound of the ratio between the value of upper bound and the value of the optimal energy consumption will be calculated and re-proven.

**Figure 5:**
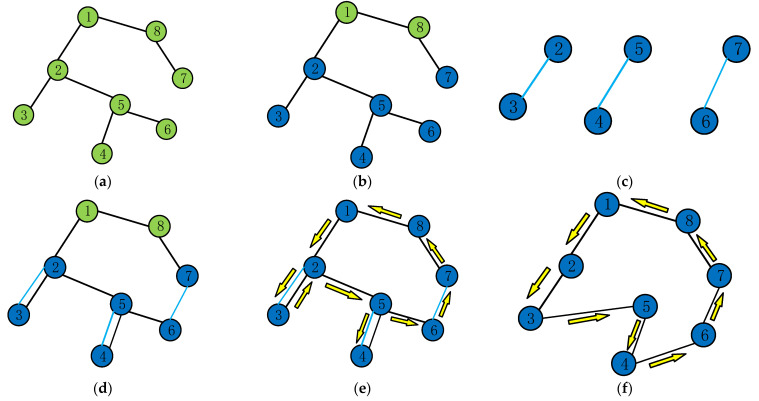
Example of Christofides’s heuristic algorithm. (**a**) Minimum spanning tree *T*, (**b**) blue points are odd degrees in set *O*, (**c**) minimum perfect matching *R*, (**d**) re-graph *I*, (**e**): Euler circuit *X* and (**f**) final path *S*.

The steps of the Christofides’s Heuristic algorithm will be briefly described as follows and its simulation process is given in Figure 5.

Step 1: For a complete graph *G = (V*, *E)*, find the minimum spanning tree *T* with the origin as the root point by the Prim algorithm (spanning tree: *m* − 1 edges, connecting *m* points in *G*, minimum spanning tree: the spanning tree with the shortest total length).

Step 2: Add all points of odd degree in *T* to the point set *O* (degree: the number of edges connecting a point).

Step 3: Construct the minimum perfect matching *R* of the point set *O* on the original complete graph.

Step 4: Take and merge the edge set *R* and edge set *T* to construct a re-graph *I*, which will satisfy that every point in graph *I* is of even degree and that graph *I* can form an Euler circuit *X*.

Step 5: Skip the duplicate points in the Euler circuit in the previous step and transform the heavy graph *I* in Step 4 into a Hamiltonian circuit graph *S*, which is the final path which we find by Christofides’s heuristic algorithm.

For the path *S* derived by Christofides’s heuristic algorithm, there are clockwise and anti-clockwise travel modes for the feeding robot, and the energy consumption of clockwise and anti-clockwise travel modes are denoted as *C*(*S_x_*) and *C*(*S_y_*), respectively. We assume that the path *S* is $\left\{e_{01},\cdots ,e_{(m-1)m},e_{m0}\right\}$, *d_i_* is the length of *e_ij_* and the weight set of the missing feed for each feed bucket is $F_{m}=\left\{q_{0},q_{1},q_{2},\cdots ,q_{m}\right\}$(*q*_0_ *= 0*). Therefore, the *C*(*S_x_*) could be expressed as follows:

(14) $$C(S_{x})=(w+Q)\sum_{i=0}^{m}d_{i}-\left[q_{1}\sum_{i=1}^{m}d_{i}+q_{2}\sum_{i=2}^{m}d_{2}\cdots +q_{m-1}\sum_{i=m-1}^{m}d_{i}+q_{m}d_{m}\right]$$

The *C(S_x_)* could be expressed as follows:

(15) $$C(S_{y})=(w+Q)\sum_{i=0}^{m}d_{i}-\left[q_{m}\sum_{i=0}^{m-1}d_{i}+q_{m-1}\sum_{i=0}^{m-2}d_{i}+\cdots +q_{2}\sum_{i=0}^{1}d_{i}+q_{1}d_{0}\right]$$

The sum of the energy consumed by the two travel modes is as follows:

(16) $$\begin{array}{l} C(S_{x})+C(S_{x})=2(w+Q)\sum_{i=0}^{m}d_{i}-\left(q_{1}+q_{2}+\cdots +q_{m}\right)\sum_{i=0}^{m}d_{i}\\ \begin{array}{cccc} & & & = \end{array}(2w+Q)\sum_{i=0}^{m}d_{i} \end{array}$$

Without loss of generality, let the *C*(*S_x_*) ≤ *C*(*S_y_*), so we can derive the following relation:

(17) $$C(S_{x})\leq C\left(S_{x}\right)+C\left(S_{y}\right)=\left(2w+Q\right)\sum_{i=0}^{m}d_{i}$$

We assume that the optimal path S^*^ is $\left\{e_{01}^{*},\cdots ,e_{(m-1)m}^{*},e_{m0}^{*}\right\}$, $d_{i}^{*}$ is the length of $e_{i}^{*}$($e_{i}^{*}\in S^{*}$), the weight set of the missing feed for each feed bucket is $F_{m}^{*}=\left\{q_{1}^{*},q_{2}^{*},\cdots ,q_{m-1}^{*}\right\}$, and the minimum value of the elements in the set $F_{m}^{*}$ is $q_{\min}^{*}$. The *C*(S^*^) could be expressed as follows:

(18) $$C(S^{*})=\left(w+Q\right)\sum_{i=0}^{m}d_{i}^{*}-\left[q_{1}^{*}\sum_{i=1}^{m}d_{i}^{*}+q_{2}^{*}\sum_{i=2}^{m}d_{i}^{*}+\cdots +q_{m-1}^{*}\sum_{i=1}^{m-1}d_{i}^{*}+q_{m}^{*}d_{m}^{*}\right]$$

If the number of feed buckets to be fed is even, there is the following relationship:

(19) $$\begin{array}{cc} C(S^{*}) & \geq (w+Q)\sum_{i=0}^{m}d_{i}^{*}-\left(\min\left\{q_{1}^{*},q_{m}^{*}\right\}+\min\left\{q_{2}^{*},q_{m-2}^{*}\right\}\cdots +\min\left\{q_{m/2}^{*},q_{m+1/2}^{*}\right\}\right)\sum_{i=0}^{m}d_{i}^{*}\\ & \geq \left(w+Q-\frac{m}{2}q_{\min}^{*}\right)\sum_{i=0}^{m}d_{i}^{*} \end{array}$$

If the number of feed buckets to be fed is odd, there is following relationship:

(20) $$\begin{array}{cc} C(S^{*}) & \geq (w+Q)\sum_{i=0}^{m}d_{i}^{*}\\ & -\left(\min\left\{q_{1}^{*},q_{m}^{*}\right\}+\min\left\{q_{2}^{*},q_{m-2}^{*}\right\}\cdots +\min\left\{q_{(m-1)/2}^{*},q_{(m+1)/2}^{*}\right\}+q_{(m+1)/2}^{*}\right)\sum_{i=0}^{m}d_{i}^{*}\\ & \geq \left(w+Q-\frac{m-1}{2}q_{\min}^{*}\right)\sum_{i=0}^{m}d_{i}^{*} \end{array}$$

The following relations could be obtained by combining Equations (19) and (20):

(21) $$C(S^{*})\geq \left(w+Q-\frac{m}{2}q_{\min}^{*}\right)\sum_{i=0}^{m}d_{i}^{*}$$

Since the maximum ratio of the length of path edge to the optimal length of path edge solved by Christofides’s heuristic algorithm is 3/2, there exists the following relationship between two lengths of paths:

(22) $$\frac{\sum_{i=0}^{m}d_{i}}{\sum_{i=0}^{m}d_{i}^{*}}\leq \frac{3}{2}$$

Combining Equations (17), (21) and (22), the relationship between the value of path energy consumption calculated by Christofides’s heuristic algorithm and the value of optimal path energy consumption could be found as follows:

(23) $$\frac{C\left(S\right)}{C\left(S^{*}\right)}\leq \frac{\left(w+\frac{Q}{2}\right)\sum_{i=0}^{m}d_{i}}{\left(w+Q-\frac{m}{2}q_{\min}^{*}\right)\sum_{i=0}^{m}d_{i}^{*}}=\frac{3\left(w+\frac{Q}{2}\right)}{2\left(w+Q-\frac{m}{2}q_{\min}^{*}\right)}$$

From Equation (23), we find the worst case of the path energy consumption derived by Christofides’s heuristic algorithm. The path consumption calculated by Christofides’s heuristic algorithm could be used as the initial upper bound.

**Figure 6:**
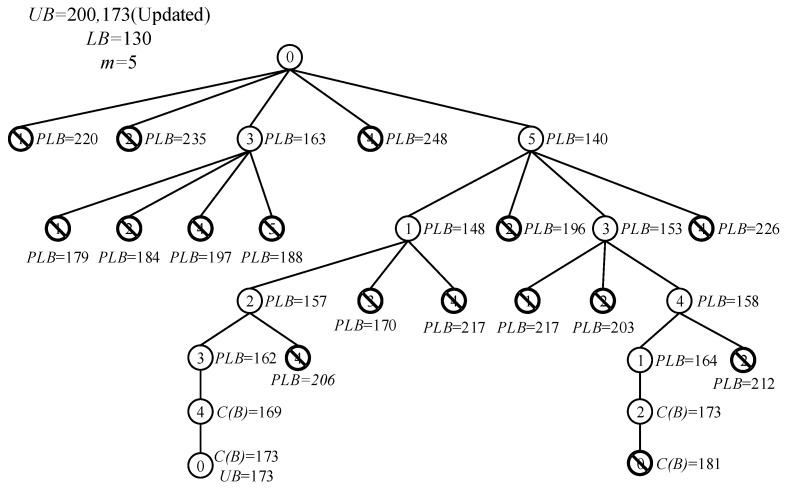
Search tree for branch and bound algorithm.

The steps of the branch and bound algorithm in this study are as follows and its simulation process is given in Figure 6.

Step 1: Calculate the initial upper bound of the energy consumption of the feeding robot path, take the root point (start point of feeding robot) as the parent point, calculate the partial lower bound of its child points, cut off the child points whose value of the lower bound is larger than that of the upper bound, and keep the child points whose value of the lower bound is smaller than that of upper bound.

Step 2: Select the child points with the shortest partial lower bound in Step 1 and repeat the operation in Step 1 until reaching the last point.

Step 3: When reaching the last point, if the energy consumption value of the complete path is less than the current upper bound value, the original upper bound value will be replaced by the energy consumption value of the complete path. Then, the new upper bound is compared with the partial lower bound of all uncut child points again; the child points whose value of the partial lower bound is larger than that of the new upper bound will be deleted. After that, branching and cutting will continue on other points, and when the value of upper bound is equal to the energy consumption value of the current complete path, the search process will stop and the path will be regarded as the optimal one. Otherwise, the optimal path will be obtained after each point has been branched and cut.

Step 4: If the energy consumption value of the complete path is larger than the current upper bound value, the current upper bound value is retained and the branching and cutting of other points will be continued. If the energy consumption value of the complete path is smaller than the current upper bound value, Step 3 is repeated.

From the above steps, the path corresponding to the shortest energy consumption of the feeding robot can be found.

### 2.3. Large Scale Feeding Point Path Planning

The problem of this study can only be solved by two types of methods: one is the exact algorithm and the other is the heuristic algorithm. With the increase in the number of feeding buckets lacking feed, the time spent by the branch and bound algorithm will be greatly increased, and it will not be able to complete the computation in the specified time. Thus, when the number of feed buckets lacking feed becomes larger, the path planning can only be performed by the heuristic algorithm. It is well known that the genetic algorithms search the optimum from a population of points in parallel and possess more chances to find the global optimum than other heuristic algorithms such as the ant colony algorithm, the cuckoo algorithm, etc. The robustness of the search process and the effectiveness of their genetic operators, such as crossover, mutation, selection, etc., show that this method is full of vitality [27]. In addition, the heuristic algorithm can only compute the approximation of the exact solution, and the accuracy of the calculation results is inferior to that of the exact algorithm. Therefore, a double-crossing operator genetic algorithm based on the upper bound of energy consumption is proposed to solve the problem in this study.

In this study, a double-crossing operator genetic algorithm based on the upper bound of energy consumption is proposed to solve the large-scale feeding point path planning. The upper bound-based double-crossing operator is used to improve the global search ability and local search ability at the same time.

#### 2.3.1. Determination of the Fitness Function

The fitness function of the genetic algorithm is criteria to evaluate the chromosome, and a well-designed fitness function will be able to reflect the individual differences more easily, which will ensure a better operation of the selection operation and prevent the early convergence condition.

In this study, what we need to solve is minimizing the energy consumption in the complete process of feeding performed by the feeding robot. Therefore, the reciprocal of the Equation (2) will be used as the fitness function, and the fitness function is as follows in Equation (24):

(24) $$f\left(B\right)=\frac{1}{d_{0}(w+Q)+d_{1}(w+Q-q_{1})+\cdots +d_{m-1}(w+Q-\sum_{i=1}^{m-1}q_{i})+d_{m}w}$$

#### 2.3.2. Double Choice Operator

At the beginning of each cycle of the genetic algorithm process, individuals are selected from the current population using the appropriate selection operator, and these individuals are used as parents of the next generation of individuals. This selection is based on probability, and the probability of being selected is related to their fitness, so individuals with higher fitness have a higher chance of being selected.

In order to improve the global search ability of the algorithm and to increase the convergence speed of the algorithm, the tournament algorithm is used first to ensure the convergence speed of the algorithm, i.e., to keep a small group of individuals with the largest fitness value. For the remaining individuals in the population, the roulette selection operator is also used to keep a small group of individuals, and it improves the global search ability of the algorithm. Roulette selection is a put-back random sampling approach, i.e., the probability of each individual entering the next generation is the ratio of its fitness value with the sum of the fitness values of the individuals in the entire population, so the individuals with higher fitness values have a higher probability of being selected.

#### 2.3.3. Double Cross Operator Based on Upper Bound on Energy Consumption

The traditional crossover operator has only one certain search advantage, either a strong global search capability or a strong local search capability [28]. In order to have both of the advantages of the operator, a double-crossing operator based on the upper bound of energy consumption is proposed. The upper bound on the energy consumption calculated in the third part is taken as the bound we currently need. For paths corresponding to fitness values larger than the upper bound, the difference between the fitness value and the minimum energy consumption value is larger, and the gene sequences corresponding to their chromosomes are also different from those corresponding to the minimum energy consumption chromosomes, so a crossover operator with strong global search capability is needed to act on the chromosomes. Conversely, for those paths corresponding to fitness values less than the upper bound value, the difference between the fitness value and the minimum energy value is relatively small, and the gene sequences corresponding to their chromosomes are less different from those corresponding to the minimum energy chromosomes, so it is only necessary to crossover chromosomes using crossover operators with strong local search ability.

This study uses real number encoding for the chromosomes. The order crossover operator OX has a better global search capability [29]; it works by randomly selecting the start and end positions in two parent chromosomes. It copies the middle region of the start and end positions of the parent1 chromosomes to the same position in the offspring1. The genes missing in the offspring1 of the parent2 chromosome are inserted into offspring1 in order, and the offspring2 is obtained in the same way as the offspring1. Therefore, when *f*(*B*) > *UB*, the OX will be used for cross operation.

**Figure 7:**
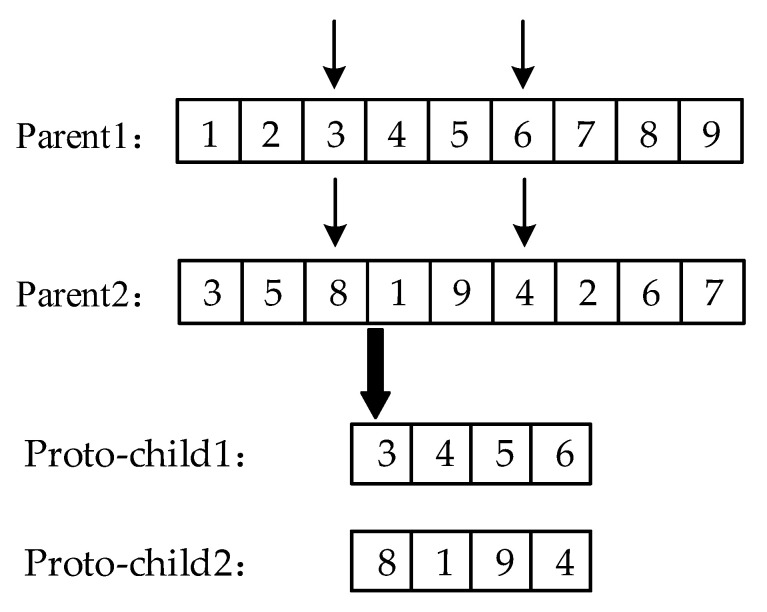
The process of extracting proto-child by Order Crossing Operator.

The OX first chooses the same starting and ending positions in both parents. Then, the middle region of the start and end positions of the two parents are extracted and copied to the two proto-children, its process of extracting proto-child is shown in Figure 7.

**Figure 8:**
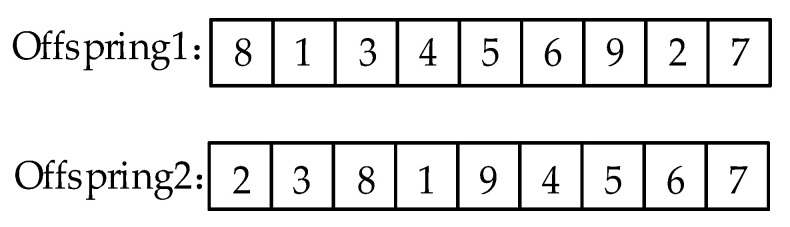
Results for order crossing operators.

Next, the missing genes from the chromosome of proto-child1 in the parent2 are filled into the proto-child1 in order, while the missing genes from the proto-child2 in the parent1 are filled into the proto-child2 in order, and the result is shown in Figure 8.

**Figure 9:**
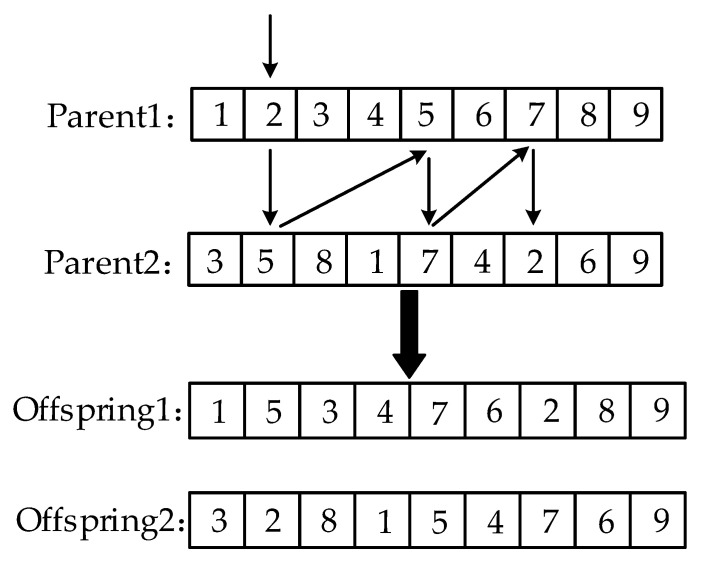
The operation process and results of CX.

The cycle crossover operator CX has a strong local search capability [30]; it works by randomly selecting a gene on one parent and finding the gene value on the corresponding position of the other parent. Then, it returns to the first parent to find the gene position with the same value, repeating the previous work until a cycle is formed. After exchanging the selected genes of the two parents, two offspring are formed. Therefore, when *f*(*B*) > *UB*, the CX will be used for cross operation. The result after the operation of CX is shown in Figure 9.

#### 2.3.4. Based on the Energy Consumption Upper Bound Mutation Operator

Mutation operators are applied to offspring after selection and crossover operations. Genetic mutations are probability-based, and usually occur with exceptionally low probability. An elevated mutation probability prevents evolutionary stagnation and ensures the diversity of the population. On the other hand, if the mutation probability is excessively elevated, the genetic algorithm will lose its usefulness to a random search algorithm, so the selection of mutation probability is particularly important [31].

In order to prevent evolutionary stagnation and to ensure population diversity, for individuals with fitness function values larger than the upper bound, the mutation probability will be set to *P*_1_ = 0.02. For individuals with fitness function values smaller than the upper bound, the mutation probability wil1 be set to *P*_2_ = 0.05.

**Figure 10:**
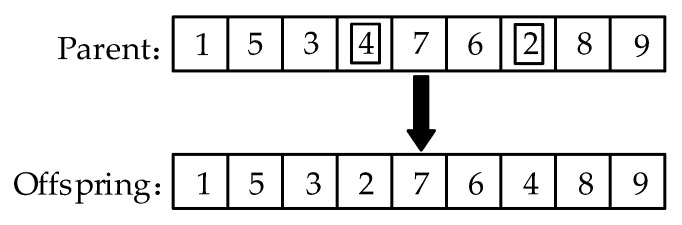
The operation procedure and results of mutation operator.

For the selection of the mutation operator, the exchange mutation is chosen as the mutation operator, which will randomly select two genes and exchange their values, the mutation process is shown in Figure 10.

#### 2.3.5. Flow of the Large Scale Feeding Point Path Planning Approach

**Figure 11:**
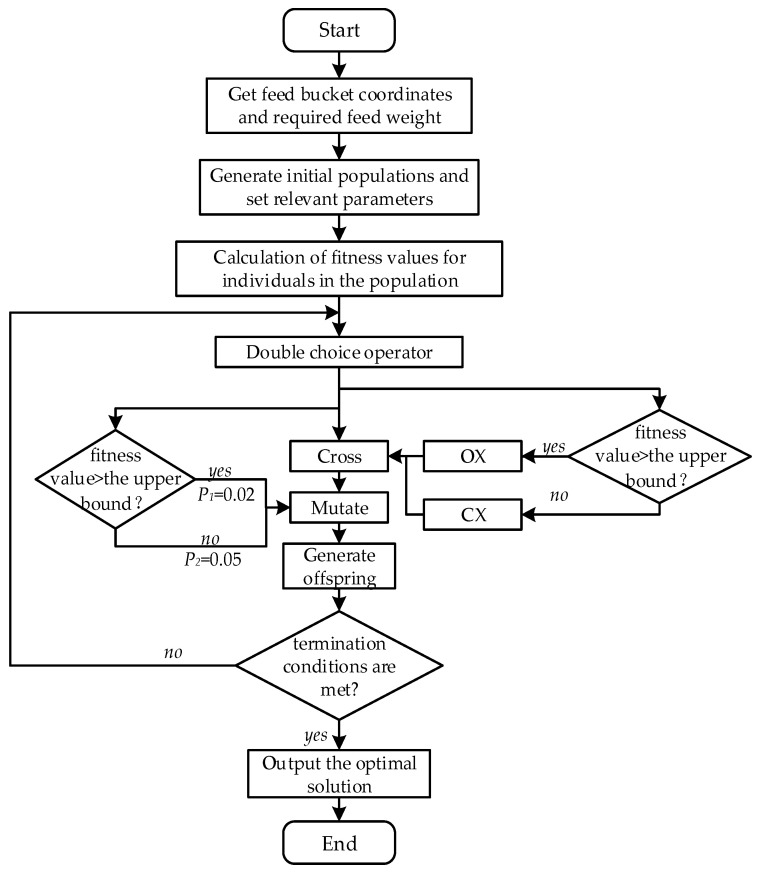
Large-scale feeding point path planning calculation process.

The steps of the double-crossover operator genetic algorithm based on an upper bound on energy consumption are as follows and its simulation process is given in Figure 11.

Step 1: Obtain feed weight information and coordinate information of the feed bucket, which are transmitted to the feeding robot through the infinite communication module of the feed bucket.

Step 2: Randomly generate the initial population and determine various parameters related to it, such as the number of selected individuals *N* = 10 and the upper bound value of energy consumption. The crossover probability is *P_c_* = 0.8, the mutation probabilities is *P*_1_ and *P*_2_ and the maximum number of iterations is *T_max_* = 1000, with a population size *S* = 500.

Step 3: *N*/2 individuals are selected from the population by the tournament algorithm, while *N*/2 individuals are selected by the roulette wheel algorithm, and the selected *N* individuals are subjected to the crossover operation.

Step 4: Perform crossover operation for *N* parents, perform order crossover for individuals with a value of fitness larger than that of upper bound, and perform cycle crossover for individuals with a value of fitness smaller than the value of the upper bound.

Step 5: The generated offspring are subjected to mutation based on the upper bound of energy consumption, and the individuals with value of fitness larger than that of upper bound are subjected to exchange mutation with a probability of *P*_1_ = 0.02. The individuals with value of fitness smaller than the value of upper bound are subjected to exchange mutation with a probability of *P*_2_ = 0.05.

Step 6: Determine whether the termination condition is satisfied: if it is satisfied, then the operation ends and the optimal solution is output; if it is not satisfied, then return to step 3 and continue iteration.

## 3. Results

According to the above algorithm steps, the algorithm was designed and implemented in Pycharm using Python 3.1. The CPU used was inter(R) Core(TM) i7-9570H with a main frequency of 2.6 GHz and 8 GB of RAM memory.

Before the sample experiment, we set the net weight of the feeding robot to 75 kg. The total number of feed buckets was 30, the horizontal and vertical coordinates of the feed buckets were randomly generated from 0–350 m, the missing weight was randomly generated from 5–20 kg, and the loading weight of the feeding robot was the total missing weight of each feed bucket to be fed on the smart farm.

Sample 1: Experimental verification of the proposed lower bound of energy consumption. When the sample experiment started, the number of feed buckets was 8, the line LB1 was the lower bound, obtained by using the approach proposed in this study, the line LB2 was the lower bound of energy consumption, obtained by the minimum spanning tree algorithm [32], and the line R was the exact result of energy consumption. The experimental results are as follows.

**Figure 12:**
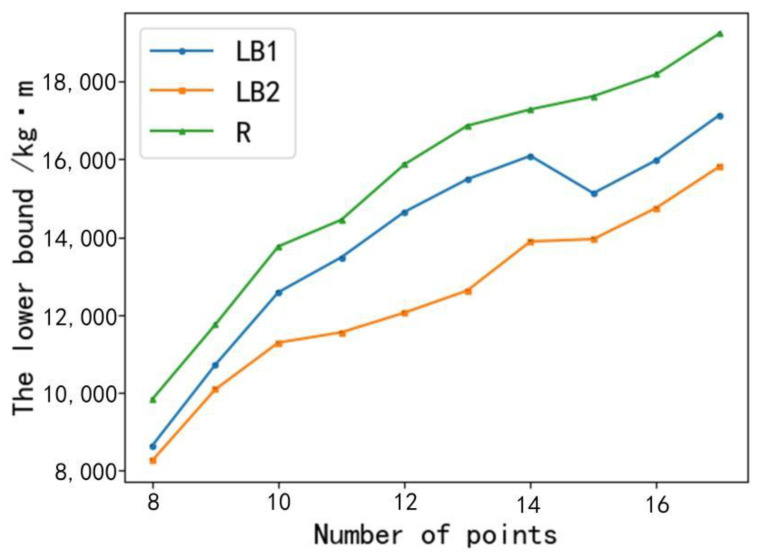
Comparison of algorithms for solving the lower bound of energy consumption.

From Figure 12, it could be seen that all the value of lower bound of energy consumption for each experiment is smaller than the final result (line R). The comparison between LB1 and LB2 shows that the experimental result of LB1 for each experiment is larger than that of LB2. For the branch and bound algorithm, the more accurate the lower bound (i.e., the larger the lower bound), the stronger the solution capability of algorithm. Therefore, the lower bound of energy consumption solved by the algorithm proposed in this study is better than the minimum spanning tree algorithm under the same parameter conditions.

Sample 2: Experimental verification of the upper bound on energy consumption proposed in this study. When the sample experiment started, the number of feed buckets was 8. Line UB1 was the upper bound of energy consumption obtained by the Christofides’s heuristic algorithm, line UB2 was the upper bound of energy consumption obtained by the greedy algorithm [33], and line R was the exact result of the energy consumption of each experimental path. The experimental results are as follows.

**Figure 13:**
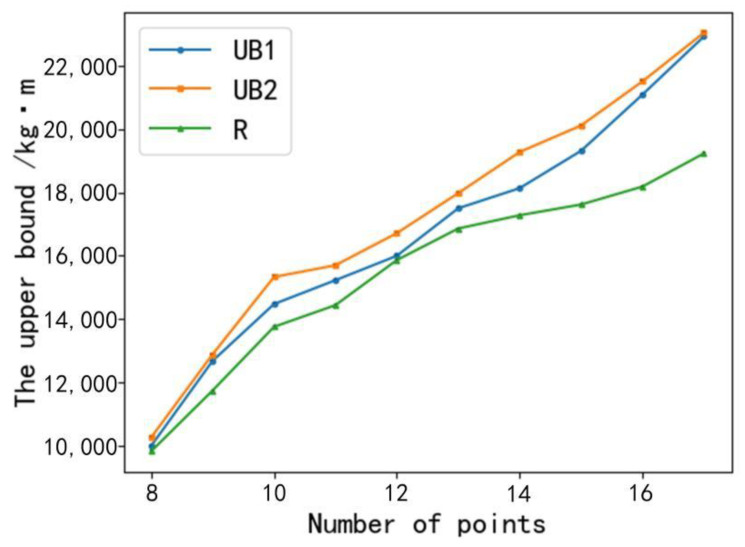
Comparison of algorithms for solving the upper bound on energy consumption.

From Figure 13, it can be seen that the final result (line R) of each experiment was less than the upper bound of energy consumption obtained by the other two algorithms. The comparison between UB1 and UB2 shows that the experimental result of each experiment of UB1 was less than that of UB2. For the branch and bound algorithm, the more accurate the upper bound of energy consumption (i.e., the smaller the upper bound), the more effective the branch and bound algorithm was in solving the problem. Therefore, the proposed algorithm for finding the upper bound on energy consumption in this study is better than the greedy algorithm under the same parameter conditions.

**Figure 14:**
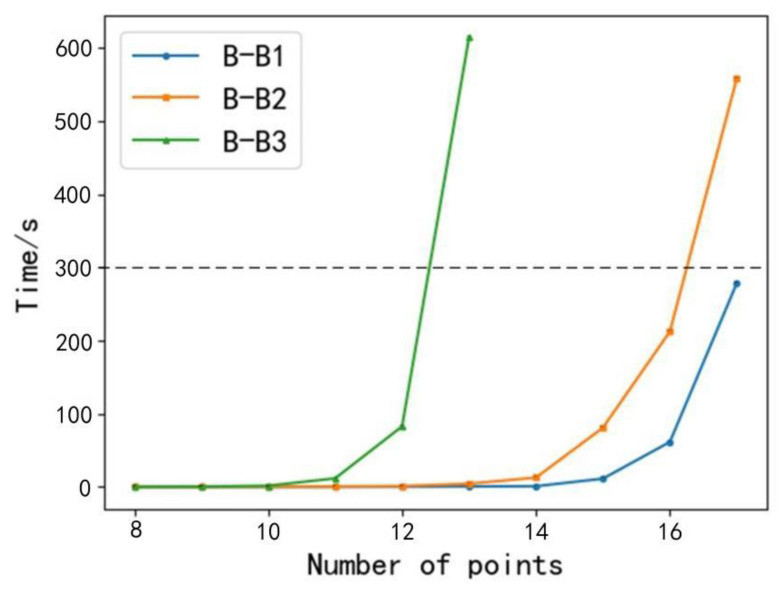
Comparison of the three branch and bound algorithms.

Sample 3: A sample experiment with three branch and bound algorithms was performed, and a comparison was made. B-B1 was the branch and bound algorithm proposed in this study, B-B2 changed the calculation method for obtaining the upper bound of B-B1 to the greedy algorithm and the rest was the same as B-B1. B-B3 changed the calculation method of obtaining the lower bound of B-B1 to the minimum spanning tree method, and the rest was the same as B-B1. The time limit of the algorithm operation is 300 s. The results of the three branch and bound algorithms are shown in Figure 14.

From Figure 14, it can be seen that the operation results of the B-B3 have a significant gap compared with those of B-B1 and B-B2. For each additional point from the second point onward, the operation time of B-B3 increased substantially, and when the number of points reaches 13, it exceeded the time limit, and the B-B3 algorithm was finally able to calculate the energy consumption of 12-points path.

The B-B1 and B-B2 algorithms took approximately the same amount of time for the initial five experiments, and the time limit was exceeded when the number of experimental computing points of the B-B2 algorithm reached 17. The B-B2 algorithm was finally able to calculate the energy consumption of the 16-points path. From the fifth point onward, the B-B1 algorithm took significantly less time than the B-B2 algorithm for each experiment, and the time limit was exceeded when the number of points of the B-B1 algorithm reached 18, so the B-B1 algorithm could finally calculate the energy consumption of the 17-points path.

From Sample 1 to Sample 3, we can conclude that the bounds of the branch and bound algorithm proposed in this study were performed more accurately; thus, when more accurate bounds are applied to the branch and bound algorithm, its computing power will also be improved, and we can verify this conjecture by the results of Sample 3. In summary, the B-B1 algorithm can produce experimental results more quickly than the other two algorithms under the same experimental conditions; thus the B-B1 algorithm is more powerful for small-scale feeding points.

Sample 4: When the number of feed buckets with a feed shortage exceeded 18, a double-crossover operator genetic algorithm based on the upper bound of energy consumption was used to perform the operation experiment, and the coordinates of the points and the weight of the feed bucket to be fed were obtained in the same way as in Sample 1.

We used three genetic algorithms as the experimental control group and compare their calculated energy consumption values. GA-1 was the double-crossing operator genetic algorithm based on the upper bound of energy consumption described in this study. GA-2 only used the order crossover operator as its crossover operator, and the rest was the same as GA-1. GA-3 only used cycle crossover operator as its crossover operator, and the rest was the same as GA-1.

**Figure 15:**
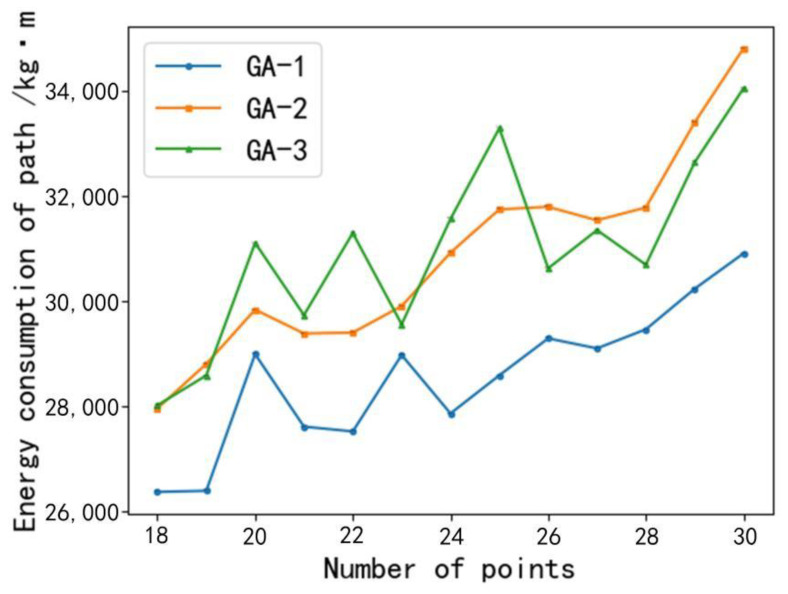
Experimental comparison chart of three genetic algorithms.

As can be seen from Figure 15, the energy consumption calculated by GA-2 and GA-3 under the same conditions does not differ much with the increase in the number of feeding points. However, the energy consumption calculated by GA-1 is much less compared with GA-2 and GA-3, so GA-3 could obtain more accurate results when the energy consumption of the path with a large scale of feeding points is calculated.

## 4. Discussion

The algorithm proposed in this study can calculate the minimum energy consumption path of different scale feed buckets lacking feed in the specified time. For small-scale feed buckets lacking feed, the minimum energy consumption travel path was obtained by the branch and bound algorithm, and the lower bound of the branch and the bound of the energy consumption was obtained by the approach of preferred selection of the set of shortest edges combined with the sequence inequality. The upper bound could be obtained based on Christofides’s Heuristic algorithm. The experiment results show that it could calculate the minimum energy consumption path of 17 points in 300 s. For large-scale feed buckets lacking feed, a double-crossover operator genetic algorithm based on an upper bound on energy consumption was proposed to calculate the minimum energy consumption travel path. The method of calculating the upper bound of energy consumption in the branch and bound algorithm was combined. Different crossover operators were innovatively proposed to perform crossover operations with the upper bound of energy consumption as the bound, so that the calculation results of the genetic algorithm could converge twice and improve the accuracy of the results. The experiment results show that it could calculate the minimum energy consumption travel path within 30 points in 60 s.

At present, most solutions to problems similar to that in this study are dominated by the length of the path and plan a path with the shortest travel length [34]. Although the method can solve the problems that appear in the paper, the scope of application of the method is limited. Compared with the method of this study, for tasks of different scales, two algorithms are not used for separate calculations. Thus, the final travel path which is sought cannot guarantee either the shortest travel path or the minimum energy consumption. Therefore, the method proposed in this study is more applicable. In the literature [35], a branch and bound algorithm is used to solve the problem of UAV delivery. It can indeed solve the path planning problem of UAV delivery, but the solution of the boundaries in the algorithm is not as accurate as the boundaries obtained in this study, and it can only plan the path of at most 15 target points. Moreover, the algorithm is still dominated by the length of the path, and the energy consumption of the UAV is not taken into account, so the efficiency of the battery energy use of the UAV in delivery does not reach the optimum. The literature [36] solves the UAV flight cost problem by building an integer programming model, which is faster for small scale target point tasks, but as the number of target points gradually increases, the solution time will increase exponentially. Compared with the method of this study, the minimum energy consumption travel path under two different conditions was obtained by two different algorithms in our study. This can reduce the production cost of feeding robot to a certain extent.

## 5. Conclusions

In this study, we described and analyzed the path planning problem for the feeding task of different scale feed buckets lacking feed in poultry smart farms. Considering the importance of energy consumption for feeding robot, energy consumption was used as the optimization objective. For small-scale feed buckets lacking feed, a branch and bound algorithm was proposed to find the minimum energy consumption travel path. The lower bound of the branch and bound on the energy consumption was obtained by the approach of preferred selection of the set of shortest edges combined with the sequence inequality, and the upper bound was obtained based on Christofides’s heuristic algorithm. Finally, we verified its efficiency through experimental simulation and found its maximum number of operations for feed buckets lacking feed. For large-scale feed buckets lacking feed, a double-crossover operator genetic algorithm based on an upper bound on energy consumption was proposed to find a path with approximation of minimum energy consumption. The different crossover operations in this algorithm were performed according to the relationship between the fitness value and the upper bound of energy consumption. In the experimental part, it was verified that the energy consumption of the path is lower than that of the single-crossover genetic algorithm by using the double-crossover genetic algorithm, based on the upper bound of energy consumption.

The method proposed in this study still has some defects: the bounds of the branch and bound algorithm are not solved with particularly high accuracy, resulting in limited computing performance of the branch and bound algorithm, and the accuracy of the upper bound on energy consumption also affects the computational efficiency of the genetic algorithm proposed in this study.

In the future, new methods will be explored in order to improve the accuracy of the bounds and to enhance the computing performance of the path planning algorithm for feeding robots in smart farms.

This paper is a study on the path planning of the feeding robots in smart farms, which also provides a theoretical basis for the path planning of the feeding robots in subsequent practical operations.

## Data Availability

Not applicable.

## Abbreviations

*G*	a complete graph representing the smart farm
*V*	the set of feed buckets in the farm
*E*	the set of edges of the complete graph *G*
*LB*	the lower bound
*UB*	the upper bound
*G_m_*	a complete graph representing the smart farm with feed buckets lacking feed
*V_m_*	the set of feed buckets lacking feed in the farm, include the starting and the final return point of the feeding robot
*V*_1_	the set of feed buckets in the farm
*E_m_*	the set of edges of the complete graph *G_m_*
*B*	a complete path of the feeding robot
*PLB*	partial lower bound
*LB_m−k_*	lower bound of energy consumption of non driving path points
*C*(*B_k_*)	energy consumption of the determined path
*V_k_*	the determined path that feeding robot has traveled
*B_k_*	the undetermined path that feeding robot has not traveled
OX	order crossover operator
CX	cycle crossover operator
LB1	the lower bound obtained by using the approach proposed in this study
LB2	the lower bound of energy consumption obtained by the minimum spanning tree algorithm
R	the exact result of energy consumption
UB1	the upper bound of energy consumption obtained by the Christofides’s Heuristic algorithm
UB2	the upper bound of energy consumption obtained by greedy algorithm
B-B1	the branch and bound algorithm proposed in this study
B-B2	change the calculation method of obtaining the upper bound of B-B1 to the greedy algorithm, and the rest is same as B-B2
B-B3	change the calculation method of obtaining the lower bound of B-B1 to the minimum spanning tree method, and the rest is same as B-B2
GA-1	the double-crossing operator genetic algorithm based on the upper bound of energy consumption described in this study
GA-2	genetic algorithm with only use order crossover operator as its crossover operator
